# Environmental Benefit Assessment of Blended Cement with Modified Granulated Copper Slag

**DOI:** 10.3390/ma15155359

**Published:** 2022-08-03

**Authors:** Qinli Zhang, Bingyi Zhang, Daolin Wang

**Affiliations:** School of Resources and Safety Engineering, Central South University, Changsha 410083, China; zhangqinlicn@126.com (Q.Z.); zhangbingyicsu@163.com (B.Z.)

**Keywords:** blended cement production, modified granulated copper slag, life cycle assessment, environmental impact

## Abstract

This study aimed to investigate the environmental impact of modified granulated copper slag (MGCS) utilization in blended cement production at a representative cement plant in China. Sensitivity analysis was performed on the substance inputs, and the life cycle impact assessment (LCIA) model was applied. A detailed comparative analysis was conducted of the environmental impact of cement production in other studies, and ordinary Portland cement production at the same cement plant. Results showed that calcination has the largest contribution impact of all the impact categories, especially in causing global warming (93.67%), which was the most prominent impact category. The life cycle assessment (LCA) result of blended cement was sensitive to the chosen LCIA model and the depletion of limestone and energy. In this study, producing blended cement with MGCS effectively mitigated the environmental impact for all the selected impact categories. Results also show a reduction in abiotic depletion (46.50%) and a slight growth (6.52%) in human toxicity. The adoption of MGCS in blended cement would therefore generally decrease the comprehensive environmental impact of cement, which contributes to the development of sustainable building materials.

## 1. Introduction

Ordinary Portland cement (OPC) is the most widely produced and utilized building material due to its advantages, including excellent workability and mechanical performance [1]. Over the past few years, cement production has surged significantly, reaching 4200 metric tons in 2021, which is projected to increase globally to 4682 metric tons in 2050 [2]. It was reported that about 1.0–1.2 tons of CO_2_ were emitted into the atmosphere for each one ton of produced OPC, which mainly came from the calcination of limestone and the combustion of fuel in the rotary kiln [3]. Meanwhile, electricity was also required to grind and mill the raw materials and clinker [4]. Scholars and policymakers have considered the application of supplementary cementitious material (SCM) to reduce the consumption of cement [5]. This research can contribute to the successful application of industrial solid wastes with high pozzolanic or hydraulic activity, such as fly ash (FA) and blast furnace slag (BFS) [6,7]. However, the increasing market demand for SCMs means that the utilization of materials with higher activity is close to saturation [8]. Therefore, it is important to develop solid wastes with low reactivity as an alternative to cement.

Copper slag is a by-product of the copper smelting process. One ton of refined copper produces 2–3 tons of copper slag, resulting in a total yearly production of 4.5 million tons, and an accumulated volume of 50 million tons [9]. Granulated copper slag (GCS) is often utilized in SCMs owing to its pozzolanic activity [10]. However, due to poor performance of blended mortars, it is important to improve the reactivity of GCS. Current approaches to achieving it include mechanical and chemical activation. Mussapyrova, et al. (2021) [11] used mechanical activation on GCS. Zhang, et al. (2020) [12] investigated the properties of alkali-activated GCS cementitious materials using sodium hydroxide (NaOH) and sodium sulphate (Na_2_SO_4_) solutions. These methods usually induce intensive energy consumption and were not very effective. Feng, et al. (2019) [13] proposed a novel method where the chemical composition and glass structure of GCS are modified by calcium oxide (CaO), resulting in an improvement in pozzolanic activity at the source (the modification procedure can be seen in Section 2.1). The result indicated that the blended cement paste with 30 wt.% of modified GCS (MGCS) gained a similar compressive strength (101.8%) to the OPC paste after 28 days of curing. This method can take advantage of heat emitted from copper smelting, thereby bringing wide economic and environmental benefits [13]. Previous studies of this approach only focused on the hydration process, the microstructure, and the mechanical properties of the blended cement, but the environmental consequences were not investigated.

Life cycle assessment (LCA), an accepted environmental assessment method, is used to evaluate the potential environmental effect of cement industries. Zhu, et al. (2022) [14] investigated the environmental effects of the resource utilization of blending GCS into cement, indicating that the total environmental impacts were mitigated by near half compared with OPC. Martos, et al. (2014) [15] found that the LCA results of the cement plant relied heavily on the initial assumptions and could be determined by a simplified calculation related to global warming potential (GWP). Supino, et al. [16] conducted an LCA analysis of Italian and German cement industries, obtaining a reduction in many environmental indicators. Kua [17] applied GCS as a replacement in concrete mixtures and evaluated the associated environmental results, finding that GCS substitution represented an environmental improvement. Even though LCA can quantitatively describe the environmental footprint of blended cement production, some issues have been not addressed. Firstly, the environmental benefits were attained at the cost of reduced performance of the blended cement, which included poor durability, strength, and workability. Secondly, existing studies generally neglected the significance of the primary data obtained directly from the cement producer, causing a high degree of uncertainty.

This paper evaluated the environmental effects of blended cement with MGCS. The LCA analysis was based on the primary data obtained from a typical cement plant in Changsha, Hunan Province, China. Since atmospheric pollution and resource consumption are the most significant of all effects, the environmental consequences associated with these two factors were the focus of this study. In order to cover ecosystems, human health, and resources, key impact categories were considered as indicators of environmental benefits, including abiotic depletion potential (ADP), global warming potential (GWP), human toxicity potential (HTP), acidification potential (AP), eutrophication potential (EP), and photochemical oxidation potential (POP). Sensitivity analyses were carried out for the input (resource and energy) and the life cycle impact assessment (LCIA) model. The LCA results in this study were compared with other studies and with the production of OPC in the same plant. Finally, engineering suggestions and future work were also proposed in detail.

## 2. Methodology

LCA is performed to evaluate environmental impact and resource usage throughout the life span of a product. An LCA is conducted through four phases as per ISO 14040/14044 [18], comprising (1) goal and scope, (2) life cycle inventory analysis, (3) life cycle impact assessment (LCIA), and (4) life cycle interpretation. The whole process of blended cement production is explained in Section 2.1.

### 2.1. Blended Cement Production

During the first stage, raw materials, including limestone, clay, laterite, and gypsum, are excavated in mining regions and transported to the cement plant by trucks. During this stage, explosives are used for blasting to mine the minerals [19]. Crude oil (diesel) and electricity are depleted for crushing and truck transportation.

The second stage is the preparation of the raw meal. The limestone, clay, and laterite are successively crushed by a hammer crusher and an impact crusher to obtain particles with a diameter particle size below 20 mm [20]. Next, all the raw materials, except for the gypsum, are placed into the raw mill at corresponding ratios and are subsequently milled to a mixture, which is called “the raw meal”. The raw meal is dried in cyclone separators at an atmosphere filled with high-temperature gas from the kiln. Electricity is the primary energy source in this process.

The third stage involves calcination. The raw meal is exposed to a high-temperature atmosphere within the kiln, creating the correct conditions for chemical reactions to produce clinker. According to Pardo, et al. [21], the dry process with a preheater and pre-calciner kiln consumes less energy compared with other procedures. Accordingly, this method was selected in this study. The reactions occurring in the kiln are described in the literature [22]. This stage can result in the most intensive energy consumption (based on raw coal and electricity) and the most pollution emissions of the entire production process [3].

MGCS preparation is a crucial phase. The original copper slag was procured from a smelter of Boliden Mineral AB, Stockholm, Sweden. This was ground in the raw mill to form GCS with a particle size lower than 0.60 mm. The CaO (99.9% pure) was supplied by Changsha Danuo Building Materials Co., Ltd., Changsha, China. The procedure of MGCS production is as follows. The GCS and CaO (20 wt.%) were charged in the furnace while the heating temperature was raised to approximately 1550 °C, at which point the mixtures were completely molten [23]. The water-quenching method was then adopted to form the MGCS. The entire process was carried out within a nitrogen atmosphere with a flow rate of 150 mL/min [24]. This procedure was conducted in the Changsha Research Institute of Mining and Metallurgy Co., Ltd., Changsha, China. The produced MGCS was eventually transported by trucks to the cement plant, which uses electricity as the main energy source.

The final stage is termed as blended cement production. The clinker that formed in the kiln was cooled down using air quenching devices, where additional heat was recovered into the pre-heater and cyclone separators [25]. The clinker was subsequently blended with gypsum to form a mixture that was ground with about 30 wt.% of MGCS at the cement mill (IEA, 2018 [26]). Finally, a fine grey powder was obtained from the cement mill, and then placed in silos.

### 2.2. Goal and Scope Definition

**Figure 1:**
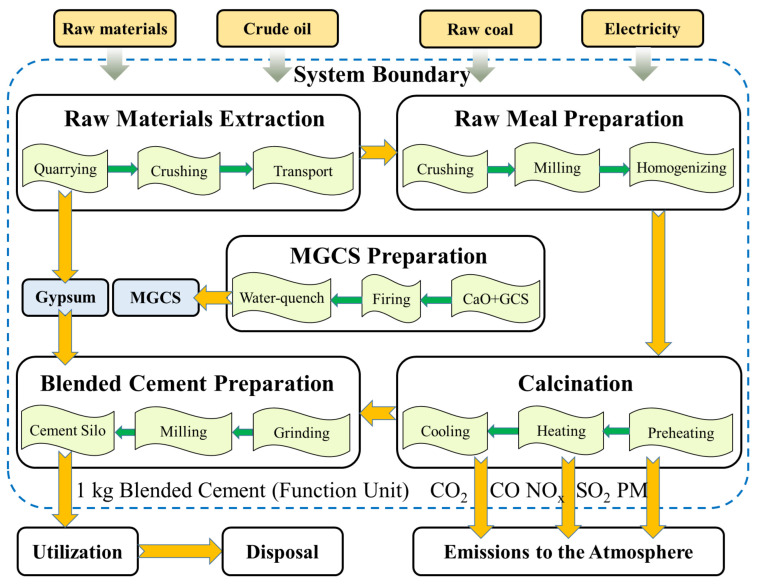
The process of blended cement production (System boundary).

As mentioned in Section 1, the purpose of this study was to evaluate the environmental benefit of using MGCS as a partial alternative to cement, based on the primary data from a typical cement plant in Changsha, Hunan Province, China. Based on the literature [27], the functional unit (FU) is defined as 1 kg blended cement with a compressive strength of 42.9 MPa. MGCS is utilized as an SCM (30 wt.%). The impact of the origin copper slag preparation was not considered in this study since it can be regarded as a waste product and is allocated no environmental burden according to EU directives [28]. Accordingly, only impacts incurred during the grinding and modification of GCS will be taken into account. The existing boundary conditions for the LCA of cement production include cradle to gate, cradle to grave, and cradle to cradle. For cradle to gate, the boundary condition is from the time that the raw materials are harvested to the time that the cement is delivered for usage. With regard to the other two cases, the LCA of the cement is performed from the moment that the raw materials are acquired from nature to the time of their disposal and reuse in a new system. This study selected the cradle to gate boundary condition. The system boundaries covered all the stages of blended cement production, and are divided into five stages, as described in Section 2.1 (Figure 1). The utilization and disposal of blended cement were not included.

### 2.3. Life Cycle Inventory

Data collection is the preparation phase of the life cycle inventory, and involves all the data associated with inputs, outputs, and energy consumption within the system boundary [29]. The inventory data of the foreground system were directly collected in the studied cement plant located in Changsha, Hunan Province, China. Some data related to the background system were obtained from the Ecoinvent 3.1 database [30]. Table 1 presents the data of blended cement and OPC related to the system boundary.

The primary inputs during blended cement production include raw materials (i.e., limestone, clay, laterite, gypsum) and energy (i.e., raw coal, crude oil, and electricity). As described in Feng, et al. (2019) [13], MGCS is utilized as a substitute with a replacement of 30 wt.% of the OPC. The energy was divided into two categories, thermal and electric. The thermal energy was generated by the combustion of raw coal and crude oil, and was consumed during calcination and transportation (by trucks). Electricity was obtained from the national grid line through thermal power and was consumed in the crushing, conveying, and grinding processes, as well as during MGCS preparation.

Apart from inputs, emissions into the atmosphere were identified as the main substance outputs in this paper. Most of the emissions resulted from the calcination phase in blended cement production, such as carbon dioxide (CO_2_), carbon monoxide (CO), sulfur dioxide (SO_2_), nitrogen oxides (NO_x_), and particulate matter (PM) [31]. Several metals (As, Hg, and Cr, etc.) were also released in low quantities into water and were therefore not considered in this paper. The transportation of the raw materials, the MGCS, and the fossil fuel depended on the distances between the producers and the cement plant and were therefore assessed as a background system [32].

### 2.4. Life Cycle Impact Assessment

The software SimaPro 9.0.0 (developed by PRé Consultants in Amersfoort, The Netherland) was used for the LCA calculation in this study. SimaPro can be directly associated with widely recognized databases including the Ecoinvent database. These databases provide a significant amount of data resources related to cement and concrete production, and are therefore commonly used in the environmental field [33]. The CML-IA baseline method that was developed by the Centrum voor Milieuwetenschappen Leiden (CML), was selected to conduct the impact assessment. The LCA model provided the results according to six impact categories: ADP, GWP, HTP, AP, EP, and POP. These impact categories influence ecosystems, human health, and resources, and cover local, regional, and global effects during the whole cement production process.

## 3. Results and Discussion

### 3.1. LCIA Results

The environmental impact of inputs and outputs during blended cement production is presented in the LCIA. The parameters of substance extraction, energy consumption, and atmospheric emission were assigned to six impact categories in the software SimaPro 9.0.0 to calculate the results.

#### 3.1.1. Characterization Results

The characterization results of each impact category are scaled and adjusted to 100% to quantify the effect of each stage on each impact category. It is however still rather difficult to determine which stage or impact category has the predominant total impact.

**Figure 2:**
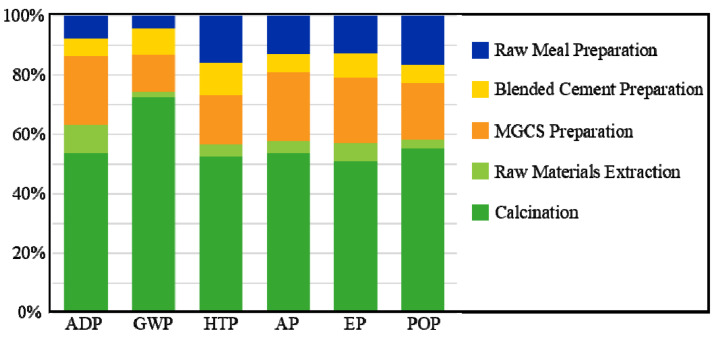
Environmental impacts of each stage to impact categories selected (characterization results).

The impact assessment model was used to calculate potential environmental effect (midpoint indicators) by the CML-IA baseline method in this study. Table 2 and Figure 2 show the characterized results (absolute values) for each stage, based on the life cycle inventory. The calcination stage generally has the largest contribution of all the impact categories (Figure 2). It should be noted that this phenomenon was expected, especially for GWP at 93.67% owing to the emissions of CO_2_ in the chemical reaction during calcination (Equation (1)):

CaCO_3_ + heat → CaO + CO_2_ (1)

The reaction shown in Equation (1) is also called “the calcination of limestone” since CaO is the main component of limestone. This reaction accounts for 67.3% of the CO_2_ emissions from the cement kiln, corresponding to 83.2% of the CO_2_ equivalent released from this system. In addition, in this stage, a significant amount of raw coal is burnt, causing CO_2_ and other greenhouse gas emissions. GWP in cement production has already been studied extensively [34]. Many measures have been proposed to reduce or avoid CO_2_ emissions, including the adoption of low-carbon alternative materials or fuels. This study adopted the latter to replace partial cement. It is important to note that 0.481 kg of CO_2_ was emitted for each 1 kg of produced blended cement, suggesting that the utilization of MGCS in cement can significantly reduce CO_2_ emissions from all the phases of blended cement production.

MGCS preparation is the second largest contributor to the majority of impact categories, accounting for 22.97% and 23.32% of the total ADP and AP, respectively.

As Figure 2 shows, calcination is the main contributor to ADP since raw materials and raw coal are sintered and consumed in this process. A large supply of electricity is required during MGCS preparation, which has a large effect on this impact category since raw coal was burnt and consumed in the thermal power plant to generate electricity.

Considering AP, massive impacts result from emissions from the cement kiln during the calcination stage, accounting for 53.55% of the total CO_2_ emissions. The second contributing factor, MGCS preparation, usually requires a mass of electricity, which was provided by raw coal. The processes of fossil fuel (raw coal) consumption and the processing of raw materials usually emit SO_2_. This SO_2_ primarily originates from the oxidation of sulfide and sulfur elements in the raw materials, or the raw coal in the presence of abundant oxygen and high temperature (usually 300 to 600 °C) [35]. AP is also affected by the release of nitrogen compounds (NO_x_) during the combustion of raw coal. As for the other phases, the preparation of raw meal represented 12.99% of AP, followed by the preparation of blended cement (6.05%) and the extraction of raw materials (4.09%), wherein raw coal is the main cause due to its consumption.

Figure 2 shows that EP is similar to AP, with calcination and MGCS preparation responsible for 50.87% and 22.20%, respectively. Regarding the last two categories, HTP and POP have a similar trend for each stage, which were primarily sourced from heavy metals emissions in coal-fired power generation plants and atmospheric emissions from all the stages. It should be noted that AP, EP, and POP are highly associated with nitrogen and sulfur compounds, as the calcination stage leads to the largest amount of gaseous emissions. POP is also affected by the carbon compounds in these emissions.

#### 3.1.2. Normalization Results

Normalization analysis was investigated since the characterized analysis cannot intuitively and directly reflect the degree of impact of each impact category on the total effect. Normalization analysis can comprehensively consider the environmental factors, and an integrated environmental impact indicator is thus obtained. The normalization result is a dimensionless value that was acquired by comparing the characterization results of each stage during the blended cement production with standardization reference indexes [36]. The total environmental impact can be calculated according to the normalization result and the weighting value.

**Figure 3:**
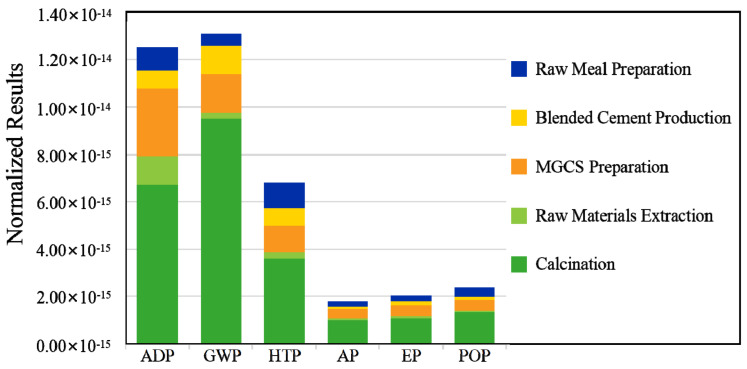
Environmental impacts of each stage to the environmental impact (normalized results).

Figure 3 and Table 3 show the results of the environmental evaluation (absolute values) for all the phases. The total environmental impact of the whole process is 3.87 × 10^−14^. The most influential process is the calcination phase that is responsible for approximately 59.78% of the total value, followed by MGCS preparation at 18.00%, raw meal preparation at 9.01%, blended cement preparation at 8.09%, and raw materials extraction at 5.12%. The largest impact of blended cement production on the environment was GWP (33.92%), followed by ADP and HTP, accounting for nearly 32.33% and 17.66% of the total, respectively. The other impact categories, including POP, EP, and AP, were 6.14%, 5.28%, and 4.67% respectively.

The above analysis indicates that the main factors influencing emissions include GWP, ADP, and HTP, which was in agreement with Yang et al., 2022 [37]. In this case, GWP and ADP were the most significant of all the indicators. Care should therefore be taken to reduce atmospheric emissions and resource consumption, especially during the calcination stage. Options that could be adopted include finding low-carbon raw materials and the installation of emission reduction devices, which are discussed in detail in Section 3.5.

### 3.2. Sensitivity Analysis

The life cycle impact assessment (LCIA) of blended cement production depends on various factors. It is imperative to carry out a sensitivity analysis to explore the key factors that result in major environmental effects. Sensitivity analysis is usually regarded as a systematic analytical approach to investigating the effect on representative system indicators when certain changes occur in the correlation coefficients, which can provide evidence for scientific and reliable decision-making [38]. The analysis results in this study are usually influenced by two indicators, the substance input parameter and the LCIA model parameter.

#### 3.2.1. Sensitivity Analysis for Substance Input Items

As it is well known, output items are heavily reliant on inputs. Sensitivity analysis was performed on the resource and energy input values to assess the significance of these inputs to LCIA analysis. Based on the literature [39], the variation coefficient of the independent variable is set at 5%, namely, the input parameters of each key process were improved or reduced by 5% in this paper, and changes in the contribution to impact categories can be calculated to explore the significance of each input parameter.

Each selected input item, i.e., limestone, MGCS, raw coal, and electricity, was changed by 5%, which changed the obtained LCIA results by 1.47%, 0.14%, 1.56%, and 1.29% respectively (Table 4). The relative change rates were calculated as 29.4%, 2.8%, 31.2%, and 25.8% respectively.

The results demonstrate that the potential environmental impact was sensitive to the consumption of limestone, raw coal, and electricity but insensitive to MGCS. These results may be attributed to two aspects. Firstly, limestone consumption mainly occurs in the calcination stage and induces a mass of greenhouse gas emissions, including CO_2_. Both raw coal consumption and electricity generation can emit a significant amount of greenhouse gas owing to combustion and thermal power generation. It is therefore important to minimize the consumption of limestone materials, which is identified as the first countermeasure to enhancing the environmental benefits of blended cement. Then, with regard to energy, it is necessary to enhance the utilization efficiency of coal and electricity and/or develop low-carbon energy transition. Additionally, pollutants emitted from the combustion of raw coal have to be discharged after being properly disposed to markedly decrease its impact on the environment.

#### 3.2.2. Sensitivity Analysis for the LCIA Model

The LCA results for implementation rely on many factors, such as the FU and the LCIA models. The LCA varies strongly from region to region, especially for resources and energy. A large amount of raw coal is produced every year in China, differing significantly from many other countries [40]. Basing the LCIA model for ADP on global data maybe therefore not fully reflect the Chinese case. Habert et al., 2012 [41] stated that the depletion of resources and energy varies with the size of the chosen region. From a global perspective, the consumption of resources is negligible. From a country perspective, resource consumption is relatively low, while from a regional perspective it plays a significant role.

**Figure 4:**
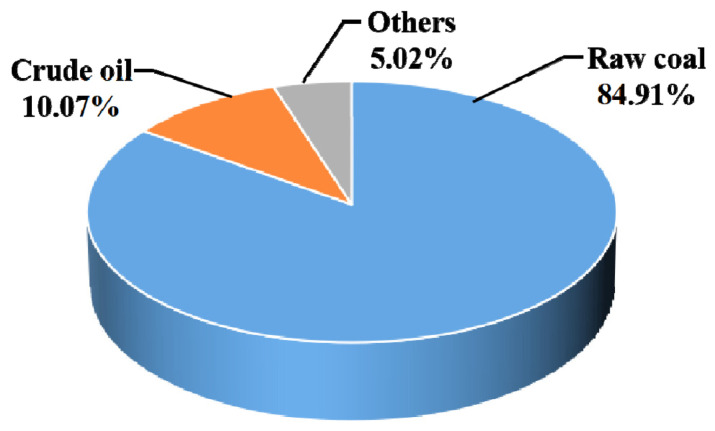
Environmental impacts to ADP of each substance in blended cement with CML method.

**Figure 5:**
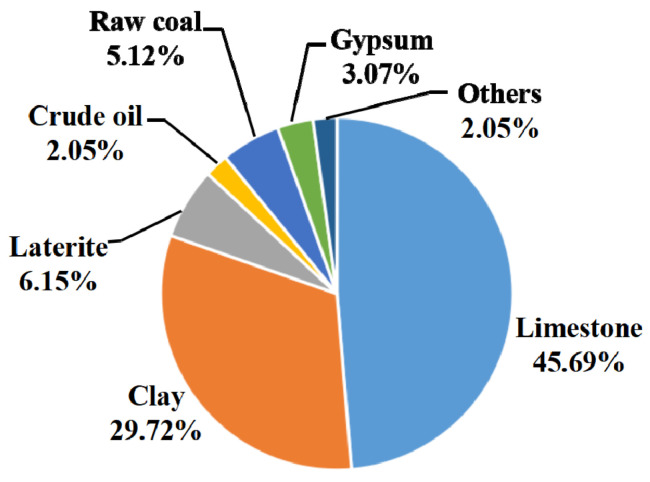
Environmental impacts to ADP of each substance in blended cement with Chinese data.

A sensitivity analysis was performed in this Section to determine the effect of different LCIA models (the CML and the Chinese method) on the results. Figure 4 and Figure 5 show the characterization results for the impact category ADP in blended cement production for the two methods. Raw coal contributes the most to ADP using the CML method, accounting for nearly 85% of the total ADP (Figure 4). In comparison, raw coal accounts for less than 10% of the total ADP with the Chinese method (Figure 5). This result can be explained by the abundant coal resources in China, causing a much lower characterization factor for raw coal when ADP is calculated using Chinese data (such as excavation and reserve amount, among others). The Chinese-based model associated with ADP usually quantifies the abiotic resource depletion potential, due to non-metal ore consumption. Figure 5 shows that limestone accounts for a large impact on ADP with the Chinese model. This is mainly due to China’s higher cement production, accounting for more than 60% of the worldwide yield, and therefore resulting in larger excavation and consumption volumes for limestone. Based on Gao et al. (2009) [42], the excavation and reserve amounts of limestone were 4.45 × 10^8^ ton and 6.59 × 10^10^ ton per year, respectively. The sensitivity analysis above therefore illustrates a marked discrepancy for the impact category ADP as calculated by the CML compared to the Chinese data.

### 3.3. Comparison with Other Studies

In this Section, comparison analyses were carried out with two similar LCA studies related to cement production in other countries.

The system boundaries, production procedures, and applied technology vary widely for the different LCA results of cement production. Some important differences should be discussed and compared with other LCAs studied in corresponding cement production processes. For example, the environmental consequence of OPC production in France was investigated using the CML-IA baseline method by Chen, et al. (2010) [43] (FU: 1 kg of cement). Input data were obtained from a cement manufacturer in France and the output data were identified as the average value collected from 15 cement plants with similar production procedures to our study. The comparison between Chen, et al. (2010) [43] and this study was then performed.

Lower impacts were generally found in all categories for our present study. The ADP is considerably lower than that of Chen, et al. (2010) and was predominantly caused by natural resources consumption. Most of this impact category can be attributed to fossil fuel (raw coal) consumption, which is used both to provide heat and to achieve electricity generation. The ADP in our study was 72.3%, against nearly 90% in Chen, et al. (2010).

GWP is also the largest contributor of the total impact categories, accounting for 70.49% of the total. According to Chen et al. (2010), emissions from the kiln account for 88.6% of GWP. This value is almost equivalent to the calcination phase, with a value of 72.51% for our study. A lower GWP value was acquired in this study (0.481 kg CO_2_ eq) when compared with that found by Chen, et al. (2010) (0.782 kg CO_2_ eq). These results imply that using MGCS as a substitute can effectively and sustainably decrease greenhouse gas impact.

For HTP, our investigation yielded a lower value compared to approximately 60% in Chen, et al. (2010). The latter study indicated that primary fuel production and direct emissions from the kiln contributed to almost the entire HTP in nearly equal contributions (nearly 50%). The calcination and MGCS preparation stages are however responsible for 52.58% and 16.63%, respectively. Blended cement production in this study therefore requires a smaller quantity of raw coal, achieving reduced combustion of fuel and lower releases of heavy metals.

With regard to the impact categories AP, EP, and POP in the Chen study, emissions from the cement kiln (atmospheric emission during the calcination stage) were identified as the main contributing process. Electricity generation is the second most important contributor due to raw coal combustion, resulting in SO_2_ and NO_x_ emissions, among others. Hence, it is natural that fewer atmospheric emissions and less fuel consumed can cause lower environmental impacts for these three impact categories in this study (Table 5).

Another meaningful LCA investigation has been conducted by Valderrama et al. (2012) [44] at a cement factory in Spain. One difference between their study and ours is that the FU for their study is 1 kg of clinker instead of cement. Their results are therefore not directly comparable with ours. Similar to Chen, et al. (2010) [43], no alternative material was utilized. The selected available techniques however played a crucial role during cement production, which achieved a reduction of 14% for ADP, 5% for GWP, 15 and 17% for AP and EP, respectively, as well as 10% for POP. The impact category HTP was not considered. They stated that energy efficiency in the cement kiln was markedly enhanced, in that a lower amount of fossil fuel was consumed, which resulted in fewer atmospheric emissions. The values of these impact categories (ADP, GWP, AP, and EP) are larger than those of our study, although these two studies corresponded to different FUs (1 kg of clinker versus blended cement). This means that the production of blended cement with MGCS can gain better environmental benefits.

**Figure 6:**
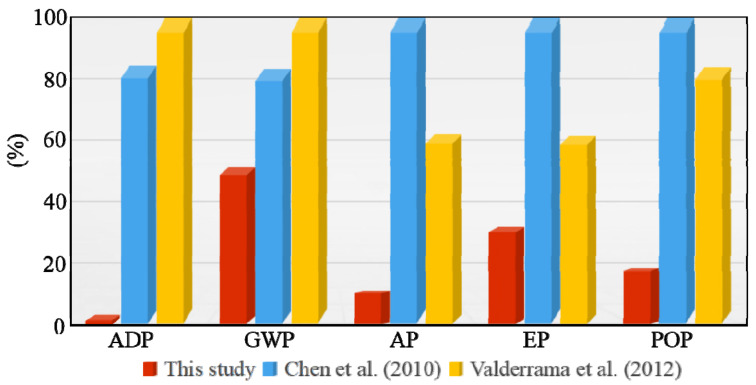
Comparison of LCA results for cement production studies [43,44].

The absolute values for several impact categories in the present study were compared to two other studies (Figure 6). Using MGCS according to the procedure proposed in this paper can significantly decrease atmospheric emissions, as a result of lower environmental impacts for all the selected impact categories. Other benefits, including the lower cost of cement manufacturing (mainly due to the usage of solid wastes), better resource conservation (mainly due to lower amount of raw materials and energy used), and the effective and sustainable treatment of GCS are also obtained, achieving a triple win [45].

### 3.4. Comparison of Environmental Impact between Blended Cement and OPC

#### 3.4.1. Comparison of Resource and Energy Depletion

Resource and energy depletion were compared between blended cement and the OPC within the FU to evaluate the environmental benefits of blended cement production. The consumption of limestone is 0.92 kg, which is lower than that of OPC (1.02 kg) by nearly 10% (Table 1). Clay in OPC is almost fully substituted by MGCS in blended cement, whilst laterite consumption is also reduced. The production of blended cement with MGCS decreased the depletion of limestone and other resources, resulting in lower energy consumption and lower atmospheric emissions during the mining, grinding, and calcination processes. The energy use of the blended cement and OPC were approximately 4.49 × 10^3^ kJ and 5.21 × 10^3^ kJ, respectively, achieving a 16% energy saving. The reasons are as follows. First, the decreased grindability of blended cement owing to the lower grindability of the MGCS consumes more electricity during grinding and milling. Second, fewer used clinkers due to MGCS addition leads to a lower quantity of raw coal depletion for calcination [46]. Considering these two aspects, the energy consumption of blended cement production can be reduced by 16% in comparison to OPC production.

#### 3.4.2. Comparison of LCIA Results

**Figure 7:**
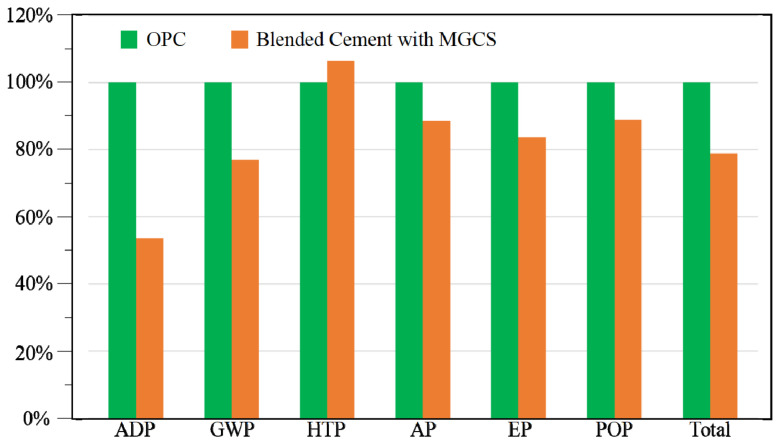
Comparison of LCA results between OPC and blended cement.

**Figure 8:**
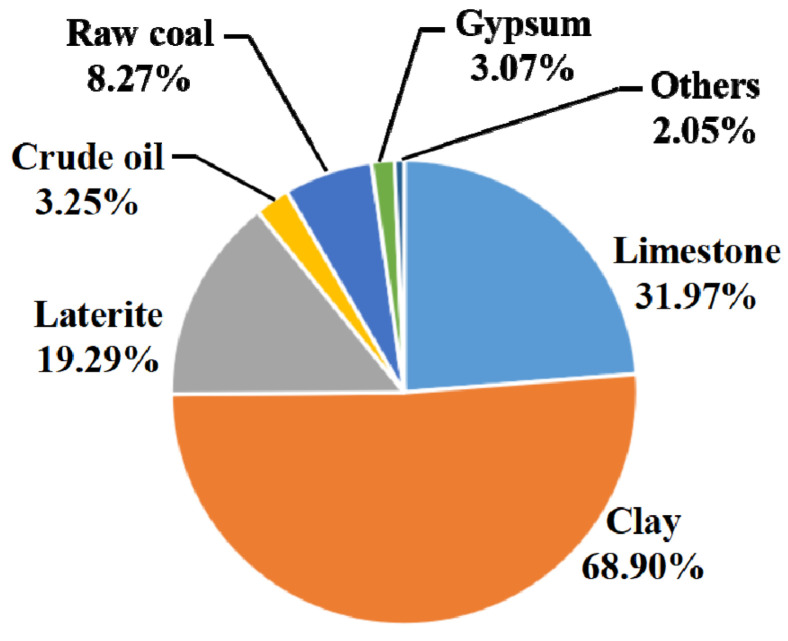
Environmental impacts to ADP of each substance in OPC with Chinese data.

Figure 7 and Table 1 illustrate the environmental consequences of blended cement with MGCS compared with that of OPC production at the same cement plant. Compared with OPC production, blended cement production with MGCS can significantly mitigate environmental burdens, especially for ADP (46.50%). Figure 5 and Figure 8 show the contributions of each material in the OPC and in blended cement to ADP. Based on the discrepancy between the CML and the Chinese data (see Section 3.2.2), the contribution of each substance to ADP is generally according to the Chinese data. In Figure 5 and Figure 8, the clay is the most important contributor to ADP for OPC and was responsible for 68.90% of the total, followed by limestone and laterite. Limestone is the predominant contributor to ADP for blended cement due to the partial MGCS substitution, resulting in a considerable reduction in the ADP of blended cement compared with that of OPC (Figure 7).

The impact category HTP of blended cement was also 6.52% higher compared to that of OPC (Figure 7). This can mainly be ascribed to the extra electricity input during the sintering of the MGCS, and the poor grindability of the MGCS during materials milling, inducing more power depletion. The HTP is primarily sourced from heavy metals emissions in coal-fired power generation plants. The total environmental impact of blended cement decreased by 13.95% compared with that of OPC, implying that the production of blended cement can not only increase the benefits of materials conservation but can also reduce the environmental impact of cement [47].

### 3.5. Engineering Recommendation and Future Work

With the continuous development of urbanization, environmental damage has become increasingly severe and has received widespread attention globally. Cement manufacturing in China induced a large amount of pollution emissions in recent years. The increasing demand for cement in building materials, accompanied by underdeveloped technology and management during production processes, has resulted in a negative impact on the environment. Based on these issues, investigating the lifespan of blended cement production in this study can contribute to grasping the key environmental indicators and point the way towards cleaner production.

In this Section, some suggestions are provided based on two aspects. The cement plant in this paper applied MGCS to replace partial OPC (30 wt.%) at an individual factory level, where raw coal is the main fuel used to provide energy. Though the contribution of raw coal is merely 5.12% in China, it represents the main energy source in many countries. According to Son et al., 2022 [48], it is imperative to improve and optimize energy composition. Natural gas is currently considered to be an eco-friendly and economical fuel. The combustion of natural gas (equivalent) causes significant reduction in atmospheric emissions, including CO_2_, CO, SO_2_, NO_x_, and PM emissions, compared with raw coal. A fuel shift that contains raw coal and natural gas can result in lower environmental impacts for many impact categories and should be further investigated in the future [49]. Meanwhile, Figure 7 indicates that limestone depletion accounts for nearly 46.00% of the total ADP in China. Considering higher sensitivity of limestone (see Section 3.2.1), it is important to develop low-cost materials with properties comparable to limestone in order to reduce its consumption.

Furthermore, technologies that reduce pollution should be further improved. Considering the cement plant in our study, four bag filters in the dust-catcher applied during the production of blended cement can prevent and reduce PM emissions to the atmosphere [50]. More bag filters can be added to achieve even lower PM emissions. Although the installation of more bag filters is a positive measure, further research and optimization should be performed in terms of emission control options, such as using desulfurization and denitrification technologies.

Nationally, further reduction of environmental effects during cement production should be assessed, even though the reuse of MGCS has already reduced many impact categories in this study. Over the past few years, China has been advocating a green and low-carbon production option for the cement industry [51]. The national government should regulate the industry’s environmental impact and provide incentives for applying pollution reduction technologies [52]. Stronger assessments and better detection of potential environmental impact are therefore required. Among these are actions to modify and improve the environmental impact assessment procedures in a country. This will ensure that potential effects are accurately monitored and that solutions to mitigate pollution are implemented [53]. Proper economic incentives for the application of pollution mitigation as well as increased use of natural gas in the fuel mix of a cement company, could assist in achieving this goal.

Finally, it is important to develop a novel low-carbon alternative to cement in the solid waste field, which can reduce emissions into the atmosphere and attain lower values for many impact categories (ADP, GWP, HTP, AP, EP, and POP). In this paper, GCS was modified by CaO under high-temperature conditions to improve its pozzolanic activity, making it a high-performance cement alternative [54]. The LCA results showed that the production of blended cement with MGCS can substantially alleviate many potential environmental effects [55]. Therefore, switching from conventional raw material to industry solid waste can be further studied. Even though the utilization of cement substitutes is an environmental option, some aspects cannot be ignored, such as operational cost and availability. Chemical components of alternative materials should be carefully detected to understand whether they contain hazardous substances. If so, the content has to be accurately monitored. Further investigations are necessary to verify the feasibility of these proposed suggestions.

## 4. Conclusions

This paper evaluated the environmental impact of replacing partial OPC with MGCS by means of the LCA methodology. Sensitivity analyses of inputs and the LCIA model used were performed. A comparative analysis of the environmental consequences of cement production in other studies and OPC production was investigated. The conclusions of this study are as follows:

1. Calcination had the largest contribution in all impact categories, significantly promoting GWP (93.67%) and the other categories.
2. Using MGCS as a replacement for cement led to lower CO_2_ emissions and had a positive effect on all the impact categories. The intensive depletion of raw materials and raw coal mostly affected ADP. The burning of raw coal contributed significantly to AP and EP. Due to the fossil fuel usage during its generation, electricity was a non-negligible contribution to AP.
3. The environmental effect of the production of blended cement was sensitive to the consumption of limestone and energy, as well as the selected LCIA model.
4. Comparisons between LCA studies show that the production of blended cement with MGCS can markedly decrease environmental impacts for all impact categories. Positive mitigation effects were obtained for most impact categories in comparison to OPC production. The adoption of MGCS in blended cement can therefore reduce the comprehensive environmental impact (13.95%).

Using MGCS as an SCM contributes to the treatment of copper slag, and thus promotes landfill reduction. The production of blended cement with MGCS can improve both waste treatment and the ‘energy-saving and emission-reduction’ of the cement industry. Further studies on the availability of natural gas utilization in fossil fuel (replacing raw coal) and emission control systems are recommended. Most importantly, it is imperative to develop more sustainable alternatives to cement to further reduce the environmental burden caused by cement production.

Of course, for copper slag it is also important to further explore solutions for excluding the toxic substance in copper slag during the process of blended cement production. Besides, copper slag has been widely utilized elsewhere, including in the manufacturing of concrete aggregate and functional materials (glass–ceramic and catalyst). Accordingly, environmental effects of related production processes should be further considered and investigated in the future.

## Figures and Tables

**Table 1:** Life cycle inventory of blended cement and OPC based on the defined FU.

Inputs	Unit	Amount (Blended)	Amount (OPC)	Outputs	Unit	Amount (Blended)	Amount (OPC)
**Raw Materials Extraction**							
Explosives	kg	2.73 × 10^−4^	3.90 × 10^−4^	PM	g	2.61 × 10^−3^	3.73 × 10^−3^
Crude oil ^a^	L	1.13 × 10^−3^	1.61 × 10^−3^	CO	g	1.22 × 10^−4^	1.74 × 10^−4^
Electricity ^b^^,c^	kWh	1.09 × 10^−2^	1.56 × 10^−2^	NO_x_	g	5.25 × 10^−3^	7.50 × 10^−3^
				SO_2_	g	1.09 × 10^−4^	1.56 × 10^−4^
**Raw Meal Preparation**							
Limestone	kg	9.20 × 10^−1^	1.02 × 10^0^	PM	g	2.84 × 10^0^	4.06 × 10^0^
Clay	kg	1.32 × 10^−2^	1.86 × 10^−1^	CO_2_	g	1.86 × 10^−3^	2.66 × 10^−3^
Laterite	kg	4.38 × 10^−2^	6.26 × 10^−2^	CO	g	1.05 × 10^−5^	1.50 × 10^−5^
Crude oil ^a^	L	1.01 × 10^−3^	1.44 × 10^−3^	NO_x_	g	1.84 × 10^−5^	2.63 × 10^−5^
Electricity ^b^^,c^	kWh	1.87 × 10^−2^	2.67 × 10^−2^				
**MGCS Preparation**							
CaO	kg	5.92 × 10^−2^	/	PM	g	3.65 × 10^−1^	/
GCS	kg	2.96 × 10^−1^	/				
Electricity ^b,c,d^	kWh	1.55 × 10^−1^	/				
**Calcination**							
Raw meal	kg	9.77 × 10^−1^	1.27 × 10^0^	PM	g	5.21 × 10^−4^	7.44 × 10^−4^
Raw coal	MJ	4.49 × 10^0^	5.21 × 10^0^	CO_2_	g	5.91 × 10^−1^	8.44 × 10^−1^
Electricity ^b^^, c^	kWh	4.21 × 10^−1^	6.01 × 10^−1^	CO	g	3.22 × 10^−4^	4.60 × 10^−4^
				NO_x_	g	1.58 × 10^−3^	2.26 × 10^−3^
				SO_2_	g	1.77 × 10^−4^	2.53 × 10^−4^
**Blended Cement Production**							
Clinker	kg	6.57 × 10^−1^	9.38 × 10^−1^	PM	g	1.69 × 10^−5^	2.41 × 10^−5^
Gypsum	kg	4.73 × 10^−2^	6.76 × 10^−2^				
MGCS	kg	3.10 × 10^−1^	/				
Electricity ^b^^,c^	KWh	7.22 × 10^−2^	6.83 × 10^−2^				

Note: ^a^ The calorific value of crude oil is 35.96 kJ/L. ^b^ Crude oil is mainly consumed for transportation (trucks). ^c^ Electricity is generated from thermal power, wherein raw coal consumed is considered in the system boundary. ^d^ Electricity consumption in the MGCS preparation occurs mainly during the milling and modification of GCS.

**Table 2:** Contribution of each stage on selected impact categories (Characterization).

Category	Unit	Raw Materials Extraction	Raw Meal Preparation	MGCS Preparation	Calcination	Blended Cement Preparation	Total
ADP	kg Sb eq	2.61 × 10^−8^	2.09 × 10^−8^	6.20 × 10^−8^	1.45 × 10^−7^	1.64 × 10^−8^	2.70 × 10^−7^
GWP	kg CO_2_ eq	8.90 × 10^−3^	1.99 × 10^−2^	5.98 × 10^−2^	3.49 × 10^−1^	4.37 × 10^−2^	4.81 × 10^−1^
HTP	kg 1,4-DB eq	1.69 × 10^−3^	7.06 × 10^−3^	7.39 × 10^−3^	2.33 × 10^−2^	4.92 × 10^−3^	4.44 × 10^−2^
AP	kg SO_2_ eq	1.54 × 10^−5^	4.90 × 10^−5^	8.80 × 10^−5^	2.02 × 10^−4^	2.28 × 10^−5^	3.77 × 10^−4^
EP	kg PO_4_ eq	9.91 × 10^−6^	2.03 × 10^−5^	3.55 × 10^−5^	8.13 × 10^−5^	1.28 × 10^−5^	1.60 × 10^−4^
POP	kg C_2_H_4_ eq	6.03 × 10^−7^	3.34 × 10^−6^	3.83 × 10^−6^	1.11 × 10^−5^	1.26 × 10^−6^	2.02 × 10^−5^

**Table 3:** Contribution of each stage on selected impact categories (Normalization).

Category	Raw Materials Extraction	Raw Meal Preparation	MGCS Preparation	Calcination	Blended Cement Preparation	Total
ADP	1.21 × 10^−15^	9.67 × 10^−16^	2.87 × 10^−15^	6.71 × 10^−15^	7.59 × 10^−16^	1.25 × 10^−14^
GWP	2.43 × 10^−16^	5.43 × 10^−16^	1.63 × 10^−15^	9.52 × 10^−15^	1.19 × 10^−15^	1.31 × 10^−14^
HTP	2.60 × 10^−16^	1.09 × 10^−15^	1.14 × 10^−15^	3.59 × 10^−15^	7.58 × 10^−16^	6.83 × 10^−15^
AP	7.37 × 10^−17^	2.35 × 10^−16^	4.21 × 10^−16^	9.67 × 10^−16^	1.09 × 10^−16^	1.81 × 10^−15^
EP	1.27 × 10^−16^	2.60 × 10^−16^	4.54 × 10^−16^	1.04 × 10^−15^	1.64 × 10^−16^	2.04 × 10^−15^
POP	7.12 × 10^−17^	3.94 × 10^−16^	4.52 × 10^−16^	1.31 × 10^−15^	1.49 × 10^−16^	2.38 × 10^−15^

**Table 4:** Sensitivity analysis of the main substance inputs.

Parameters	Limestone	MGCS	Raw Coal	Electricity
Consumption (kg/FU)	9.20 × 10^−1^	3.55 × 10^−1^	4.49 × 10^0^	6.53 × 10^−1^ *
Variation coefficient (input)	5%	5%	5%	5%
Change ratio (output)	1.47%	0.14%	1.56%	1.29%
Sensitivity	29.4%	2.8%	31.2%	25.8%

* Note: The unit of electricity is kWh/FU.

**Table 5:** Impact evaluation results compared with the study (Chen, et al. (2010) [43]) (CML-IA baseline method).

Impact Category	Unit	Chen, et al. (2010)	This Study
ADP	kg Sb eq	2.43 × 10^−3^	2.70 × 10^−7^
GWP	kg CO_2_ eq	7.82 × 10^−1^	4.81 × 10^−1^
HTP	kg 1,4-DB eq	7.60 × 10^−2^	4.44 × 10^−2^
AP	kg SO_2_ eq	3.49 × 10^−3^	3.77 × 10^−4^
EP	kg PO_4_ eq	5.04 × 10^−4^	1.60 × 10^−4^
POP	kg C_2_H_4_ eq	1.11 × 10^−4^	2.02 × 10^−5^

## Data Availability

All data in this paper are obtained from our experiment and are authentic and reliable. The publication of data has obtained the consent of all authors.
